# Microbiome—The Missing Link in the Gut-Brain Axis: Focus on Its Role in Gastrointestinal and Mental Health

**DOI:** 10.3390/jcm7120521

**Published:** 2018-12-07

**Authors:** Karolina Skonieczna-Żydecka, Wojciech Marlicz, Agata Misera, Anastasios Koulaouzidis, Igor Łoniewski

**Affiliations:** 1 Department of Biochemistry and Human Nutrition, Pomeranian Medical University in Szczecin, 71-460 Szczecin, Poland; karzyd@pum.edu.pl (K.S.-Z.); igorloniewski@sanum.com.pl (I.L.); 2 Department of Gastroenterology, Pomeranian Medical University, 71-252 Szczecin, Poland; 3 Department of Child and Adolescent Psychiatry, Charité Universitätsmedizin, 13353 Berlin, Germany; agata.misera@charite.de; 4 Endoscopy Unit, The Royal Infirmary of Edinburgh, EH16 4SA Edinburgh, UK; akoulaouzidis@hotmail.com

**Keywords:** gut brain axis, microbiota, functional gastrointestinal disorders, inflammatory bowel disease (IBD), adult stem cells

## Abstract

The central nervous system (CNS) and the human gastrointestinal (GI) tract communicate through the gut-brain axis (GBA). Such communication is bi-directional and involves neuronal, endocrine, and immunological mechanisms. There is mounting data that gut microbiota is the source of a number of neuroactive and immunocompetent substances, which shape the structure and function of brain regions involved in the control of emotions, cognition, and physical activity. Most GI diseases are associated with altered transmission within the GBA that are influenced by both genetic and environmental factors. Current treatment protocols for GI and non-GI disorders may positively or adversely affect the composition of intestinal microbiota with a diverse impact on therapeutic outcome(s). Alterations of gut microbiota have been associated with mood and depressive disorders. Moreover, mental health is frequently affected in GI and non-GI diseases. Deregulation of the GBA may constitute a grip point for the development of diagnostic tools and personalized microbiota-based therapy. For example, next generation sequencing (NGS) offers detailed analysis of microbiome footprints in patients with mental and GI disorders. Elucidating the role of stem cell–host microbiome cross talks in tissues in GBA disorders might lead to the development of next generation diagnostics and therapeutics. Psychobiotics are a new class of beneficial bacteria with documented efficacy for the treatment of GBA disorders. Novel therapies interfering with small molecules involved in adult stem cell trafficking are on the horizon.

## 1. Introduction

Recently, a new United Nation’s (UN) Commission goals on global mental health and sustainable development has been published [1]. This Commission is in line with other UN General Assembly and High-Level Meeting report and explores the issues of mental health from those with mental disorders to whole populations [2]. Accordingly, good mental health is viewed as fundamental to individual’s well-being and overall health. In addition, efforts are being focused on systemic and global changes in order to align mental health across all medical specialties [3,4]. In the context of the previously mentioned documents, the relationship between gut microbiota and mental health seems to be very interesting.

Intestinal microbiota represent one of the richest ecosystems in nature. Professor Rob Knight of the University of California, San Diego [5] reported that more than half of all cells in the human body are microorganisms, which are mainly bacteria as well as fungi and viruses. Until recently, the general belief was that intestinal microbes were mainly involved in digestive processes. With the advent of new molecular techniques and bioinformatics, in-depth research has unraveled the role of intestinal microbiota in a number of physiological processes [6,7]. The topic of global microbial diversity is so crucial and important to human health and wellbeing that scientists from Rutgers University-New Brunswick—In a recent issue of *Science*—Called for the creation of a global microbiota vault to protect humanity’s long-term health [8].

The role of the gut microbiome on human health is very diverse and implicated in the pathophysiology of various diseases. Its role in metabolism and obesity development has been clearly documented [9]. More recently, gut microbes have been implicated in the pathogenesis of cancer and microbial contribution in cancer treatment [10]. The pathogenesis and natural history of chronic GI, non-communicable diseases e.g., non-alcoholic steatohepatitis (NASH) [11], functional gastrointestinal disorders (FGIDs) [12], and non-GI disorders e.g., cardiovascular disease (CVD) [13] have been linked to GI tract microbes. The alterations of gut microbiota have been associated with neurodegenerative diseases as well as mood disturbance and depression [14,15,16]. In fact, mental health alterations are frequently observed in many GI diseases [17,18,19,20].

## 2. Paradigm Changer—Rome IV Criteria and FGIDs

For years, FGIDs were viewed as purely functional disorders with no scientific confirmation of a clear pathogenetic mechanism. According to Rome IV criteria, the phenotype of FGIDs results from an altered transmission of nerve and biochemical signals within the gut-brain-microbiota axis with mechanisms controlled by both genetic and environmental factors [21]. Additionally, few studies conducted in patients suffering from functional dyspepsia (FD) and irritable bowel syndrome (IBS) found alterations in small bowel microbiota. Zhong et al. [22] showed that *Actinomyces*, *Atopobium*, *Leptotrichia*, *Prevotella*, and *Veilonella* counts differ between FD and control patients. The finding was preceded by an observation that, in FD patients, gut barrier integrity is impaired and expressed as lowered transepithelial resistance, diminished expression of proteins of tight junctions, and, lastly, elevated levels of mast cells, eosinophils, and interstitial lymphocytes [23]. Giamarellos-Bourboulis reported a significant reduction in the diversity of small-bowel microbiota and the number of species [24]. Furthermore, Martinez et al. reported that the proportion of dilated junctions and intercellular distance between enterocytes in their apical part was elevated [25]. They also found that higher tryptase mRNA expression leads to overactive bowel movements and looser stool as per Bristol stool scale. Importantly, the degranulation of mast cells was found to positively affect the firing of visceral-nociceptive sensory neurons in IBS [26]. According to the new ROME IV criteria, the following factors contribute to the pathogenesis of FGIDs: (i) motility disturbance, (ii) visceral hypersensitivity, (iii) altered mucosal and immune function, (iv) altered gut microbiota, and (v) altered central nervous system (CNS). All of them are also associated with the concept of the microbiota-gut-brain axis.

The overlap of FGIDs and CNS disorders has been discussed in a few studies. It has been demonstrated that approximately one third of IBS patients suffer from depression [27]. More recently, Batmaz et al. [28] reviewed patients referred either directly to psychiatric clinics or from gastroenterology wards to psychiatrists and concluded that these patients were complaining of both GI and psychiatric symptoms. Furthermore, patients of the latter group complained more frequently of constipation, abdominal pain, and bloating and were more frequently diagnosed with psychotic disorders in comparison to those directly referred to psychiatric clinics. It is estimated that psychiatric symptoms occur in at least 36.5% of FGIDs patients [17]. Stasi et al. found that the highest prevalence of mental or spectrum disorders is in patients with functional constipation (60%) as compared to patients diagnosed with FD (52.4%) and/or functional bloating (47.6%). The most prevalent psychiatric disorder observed in FGIDs were the general anxiety disorder and panic diagnosis [17]. Furthermore, Wilder-smith et al. [29] identified both GI and CNS symptom profiles secondary to fructose or lactose ingestion.

## 3. The Emerging Role of the Microbiota-Gut-Brain Axis

Studies in animal models have shown that microbiota play an essential role in shaping the structure and function of the CNS [30]. Using sophisticated strategies for manipulating the microbiome, researchers observed the consequences of these changes one the brain and behavior. For example, it has been found out that the thickness of the myelin sheath, the length of dendrites, and the density of dendritic spines are controlled by microbiota [31,32]. A recent study by Lu et al. [33] conducted in humanized germ-free mice demonstrated that slow-growing mice presented skewed neuron and oligodendrocyte development as well as evident signs of neuro-inflammation. Social competences and repetitive behaviors are, at least in part, a reflection of the composition of intestinal bacteria [34]. These dependencies result directly from the existence of a physical and functional connection between the human digestive tract and the CNS. This concept called the gut-brain axis (GBA) with the participation of neural and biochemical mechanisms can be exploited for the development of new therapies for mental health disorders.

The CNS utilizes neural and endocrine pathways to cooperate with the gut. The sympathetic part of the autonomic nervous system and the hypothalamus-pituitary-adrenal axis (HPA) co-modulate the secretion, motility, and blood flow affecting intestinal permeability and influencing various GI disorders [35]. Gut neural signals are passed through the enteric nervous system (ENS) and the vagus nerve [36]. Biochemical information is carried out by cytokines, chemokines, neurotransmitters, and micro-vesicles [37] as well as directly by-products of gut microbiota metabolic activity, i.e., short chain fatty acids (SCFAs). Eventually, once in the circulation, these molecules influence HPA and GBA [38]. An elevated stress response may impair the psychosomatic well-being [39]. With pioneering work, Sudo et al. [40] demonstrated that gut microbiota is essential to proper stress hormones release and the restoration of intestinal ecosystem may reverse abnormal stress response. More recently, in vivo experimentation has demonstrated that stress mediators and their receptors’ expression are reduced in pathogen-free animals [41].

## 4. How the Gut/Brain Talks to the Brain/Gut

Intestinal microbiota as an integral part of the intestinal barrier controls the transport of antigens through the peri-cellular route to the *lamina propria* where the gut associated lymphoid tissue (GALT) is located [42]. The composition of intestinal microbiota can, therefore, influence intestinal barrier permeability, which guarantees the flow of molecules through the peri-cellular route to blood vessels. Gut microbes permanently train GALT to create immunity against commensal bacteria and food antigens but also to provide defense against pathogenic microorganisms [43]. During dysbiosis, due to GALT activation, effector cells and inflammatory mediators disrupt gut barrier integrity and result in elevated intestinal permeability [44]. The interaction of the intestinal barrier elements, thus, provides a physiologic and selective ability to absorb and secrete specific substances while inhibiting the translocation of microorganisms and the penetration of toxins and other harmful antigens [45,46].

The effects of increased intestinal permeability may manifest locally—In the GI tract—As well as extra-intestinally. For example, the concentration of zonulin—A protein that activates the intracellular signaling pathway leading to tight junctions [47] modulation and a marker of intestinal permeability increases in people with inflammatory and autoimmune diseases [48]. Of importance, the gut barrier resembles in structure and function the blood brain barrier (BBB) [49]. Both barriers are composed of epithelial and endothelial cells laced with lymphatic vessels, macrophages, and cellular tight junctions. It has already been suggested that both IBS and pseudomembranous colitis [50,51] are consequences of microbiota and intestinal barrier dysfunctions and these entities frequently coexist with depression [52,53].

## 5. Microbiota-Gut-Brain Axis and Susceptibility to Neuropsychiatric and Gastrointestinal Disease and Response to Therapy

The structure of intestinal microbiota is strongly influenced by diet and environmental stressors such as drugs. It seems that these factors dominate over the impact of genotype on the gut flora composition [54]. Consequently, it has been recognized that it may be the optimal marker of susceptibility to express certain clinical phenotypes and, thus, the response to pharmacotherapy [55]. Since the concept of bidirectional signaling between the gut and the brain started to evolve, scientists have made attempts to discover microbial fingerprints in neurology and psychiatry. Emerging research suggested that gut-brain axis dysfunction may be involved in the etiology of depression and anxiety, schizophrenia, addiction, and neurodevelopmental and neurodegenerative diseases as well as age-related cognitive decline [14,56,57,58,59]. Major microbiota-related alterations in particular neuropsychiatric conditions are summarized in Table 1.

Nevertheless, uninterrupted stress regulation is pivotal to mental health and altered stress response has been implicated in the origin of psychiatric diseases [58]. Numerous studies conducted in animals and humans have demonstrated that both acute and chronic stress interfere with intestinal barrier integrity and induce adverse alterations in intestinal microbiota composition. This has been confirmed in models of early-life [79] stress and prenatal stress [80]. Yarandi et al. [81] showed that water and ion in the gut might be reduced and elevated, respectively, under stressful conditions. This, in turn, impairs the physical protection of the gut barrier against both pathogenic microorganisms and nociceptive molecules. Furthermore, HPA activation, in particular corticotropin-releasing factor (CRF), showed a causative role in gut integrity disruption [82]. Elevated intestinal permeability was also found to be linked to stress-induced hypersensitivity of the rectum in animals, which were studied by means of partial restraint stress [83]. Winter et al. merged microbiome data from both animals and humans and selected bacterial genera that were either over-represented or appeared in a reduced number following stressor exposure. The first group included: *Desulfovibrio, Eggerthella, Holdemania, Turicibacter, Clostridium, Blautia*, *Anaerofilum* and *Roseburia* whereas those found in reduced numbers were: *Prevotella*, *Bacteroides*, *Mucispirillum*, *Dialister*, *Allobaculum*, *Faecalibacterium*, *Oscillospira*, *Ruminococcus*, *Dorea*, *Coprococcus*, and *Pseudobytyrivibrio* [84].

### Emerging New Concepts in the Development of Neurodegenerative and GI Disease—Gut Microbes, Innate Immunity, and Bone Marrow Stem Cells as Partners in Crime

Mood and psychiatric disorders as well as numerous GI disorders are related to chronic inflammation [85]. For example, post-infectious IBS alters GI tract motility and behavior [86]. A similar effect appears in patients with FD [87]. Behavioral alterations predominate in chronic GI inflammatory disorders [88]. Chronic inflammation of the ENS can easily affect the CNS. However, knowledge on mechanisms behind this phenomenon is still scarce. For decades, inflammation in the brain, which were recently implicated in the pathogenesis of psychiatric diseases, has been considered “sterile.” However, recent reports reveal the presence of the gut-vascular barrier (GVB), which, in structure and function, resembles the blood brain barier (BBB) and their communication is mediated via blood and bone marrow systems [89]. GVB controls the dissemination of bacteria from the gut into the bloodstream and the *Salmonella typhimirum* infection has been shown to decrease the Wnt/β catenin-inducible gene Axin2 (a marker of stem cell renewal) in the gut endothelium.

Importantly, the Wnt/β catenin signaling pathway is universally involved in trafficking (mobilization and proliferation) of stem cells deposited in adult tissues [90] including those located in GI tract [91]. Wnt/β catenin system has also been involved in the developmental control of BBB formation [92]. It has been demonstrated that various types of bone marrow-derived stem cells are mobilized into peripheral blood in patients and experimental animals in response to tissue/organ injury [93]. Examples include myocardial infarction [94], stroke [95], deep skin burns [96], and gut inflammation [97]. It has been previously shown that circulating peripheral bone marrow mononuclear cells (PBMNCs) were enriched with cells expressing mRNA of leucine-rich repeat-containing G-protein coupled receptor 5 (lgr-5), Achaete-scute complex homolog 2 (Ascl-2), Doublecortin Like Kinase 1 (Dclk-1), Male-specific lethal 1 homolog (MSL1), and B lymphoma Mo-MLV insertion region 1 homolog (BML-1). These markers are involved in the development of early intestinal lineage [97]. These circulating in PB cells could potentially be involved in reparatory mechanisms of peripheral tissues including the brain [98].

In fact, the release of very small embryonic-like stem cells (VSELs) and more differentiated neural stem cells (NSCs) from bone marrow into the peripheral blood in response to brain injury in rodents [99] and humans [95] has been well documented. Recent research indicates the involvement of other factors such as small bioactive lipids that may direct mobilization and trafficking of stem cells to injured organs [100]. Notably, release of sphingiosine-1-phosphate (S1P) correlates with the activation of the complement cascade and formation of the C5b-C9 membrane attack complex (MAC). Activation of proteolytic and fibrynolitis complement cascades. The release of cleavage fragments (e.g., C5a and desArgC5a fragments) could enhance the mobilization of stem cells from their niche in the bone marrow [101]. Moreover, these stem cells can be attracted from the bone marrow and from the intestinal epithelium in response to tumor or injured tissue derived plasma chemo-atractants such as stromal derived factor-1 (SDF-1), vascular endothelial growth factor (VEGF), zonulin, hepatocyte growth factor (HGF) or shphingosine-1-phosphate (S1P), ceramides, and extracellular nucleotides [102,103]. On the other hand, stem cells may secrete their own growth factors, cytokines, or even membrane-derived micro-vesicles that accelerate the regeneration process [104]. The previously mentioned factors have been frequently implicated in the pathogenesis of gastrointestinal and psychiatric disorders [89,105].

**Figure 1:**
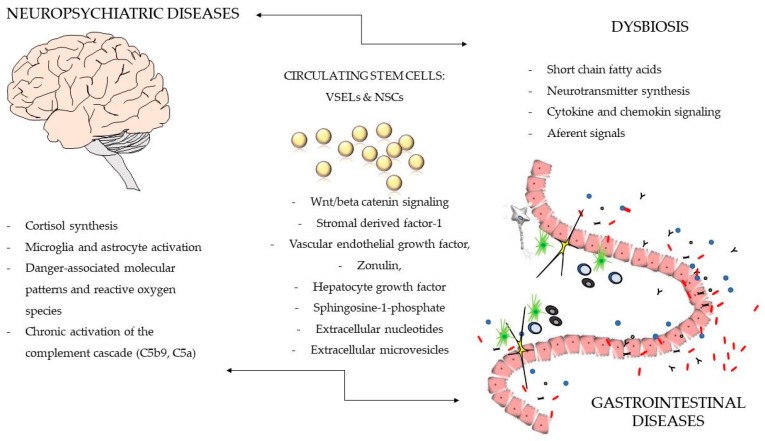
The bidirectional signaling within the GBA with the involvement of sterile inflammation of the brain and actions of microbiota and circulating adult stem cells. For details, see the text.

We envision that stem cells together with the GI microbiome are mutually involved in the pathogenesis of disorders of GBA by employing different mechanisms (e.g., autocrine, paracrine, or hormonal effects, immunomodulatory effects, replacement of damaged cells, cytotoxic effects, and neurotoxic effects) in distant tissues and organs. Further studies are needed to assess more accurately the mechanisms of cell-host-microbe interactions (Figure 1). Knowledge around these mechanisms already allows the design of novel treatments targeting GBA. For example, structural analog of S1P —Fingolimod and Ozanimod, functional antagonists of S1P receptors have already been applied in the treatment of relapsing forms of multiple sclerosis [106] as well as ulcerative colitis [107]. Similarly, supplementation with multi-species probiotics (Ecologic^®^ Barrier, Amsterdam, The Netherlands) in a 12-week, placebo-controlled, randomized clinical study, which favorably modified both functional and biochemical markers (e.g., VEGF) of vascular dysfunction in obese postmenopausal women [108].

Emerging scientific data show that rapid changes in diet and lifestyle significantly contribute to the weakening of old evolutionary cell door-keeping mechanisms (e.g., Wnt/β catenin system) in the gut and the brain. The notion that even minor and subclinical stimuli to the gut mucosal and vascular barrier (e.g., infection) can result in significant, though delayed, consequences that may seriously affect the health of an individual is attractive [109], it still requires further detailed studies.

## 6. Drug-Microbiome Interactions—Still Neglected Problem in Clinical Medicine

Although microbiota alterations play—At least in part—A role in the etiology of neuropsychiatric diseases. Paradoxically, the treatment of these conditions may adversely affect the composition of intestinal microbiota. In fact, multiple drugs were found to be involved in dysbiosis origin [110,111,112]. Recently, Maier [113] reported that approximately one-fourth of about 10,000 non-antibiotic drugs were found to be able to reduce the in vitro growth of particular bacteria strain. Among these, the psychiatric drugs were predominant. In fact, certain pharmaceuticals utilized in neurology and psychiatry, which are predominantly antidepressants and antipsychotics, were historically characterized for being antibacterial agents. Evidence gathered mostly from animal studies but also in humans suggests that second-generation antipsychotics (SGA), which are mainly olanzapine and risperidone, change the composition of intestinal bacteria toward bacterial species promoting obesity. As demonstrated by several authors, the administration of these psychotropic drugs may increase the *Firmicutes*/*Bacteroidetes* ratio [114,115,116,117,118] previously found to be a microbiota profile of the obese [119].

The majority of studies aimed to look for the link between antipsychotic-induced metabolic malfunctions and microbiota dysbiosis were in vivo models. In rats treated with olanzapine (OLZ) and mice fed with a high fat diet and administered with OLZ, the microbiome diversity was found to be reduced [115,118]. When treated with another neuroleptic agent, namely risperidone (RIS), fewer operational taxonomy units (OTUs) were found in female mice [117]. Davey et al. demonstrated gender-dependent elevation of *Firmicutes* abundance and a decrease of *Bacteroidetes* [115,116]. Antipsychotic-driven dysbiosis in rodents resulted in decreased counts of beneficial *Actinobacteria* and *Proteobacteria* [115]. The OLZ regimen introduced higher abundance of *Erysipelotrichia* and *Gammaproteobacteria* and lower abundance of *Bacteroidia*. Lastly, OLZ suppressed the growth of anaerobic bacteria [117] and *Escherichia coli* NC101 [118].

In parallel, very few studies in humans explored the alterations of the gut microbiome following SGA treatment. Skewed intestinal microbiota following the psychotropic pharmacotherapy in humans expressed as elevated phylogenetic diversity and evaluated by means of PcoA of unweighted UniFrac distances [120] and reduced Simpson diversity in females [121] have been reported. Chronic use of RIS in children elevated the levels of *Clostridium*, *Lactobacillus*, *Ralstonia*, and *Eubacterium*. This occurred only in patients with a significant gain in BMI [120]. Flowers et al. conducted gut microbiota analyses in adult patients diagnosed with bipolar disorder and demonstrated that psychotropic treatment increased concentration of family *Lachnospiraceae* in the whole cohort of patients treated with SGA and a group of obese subjects. In addition, lowered counts of *Akkermansia* genera were noticed in the whole cohort of patients receiving treatment [121]. Yuan et al. lowered the levels of *Clostridium coccoides* Kaneuchi and *Lactobacillus* spp. and elevated the numbers of *Escherichia coli* Castellani and Chalmers in adult schizophrenia patients since six weeks of RIS treatment, which demonstrated that these variations may have induced body weight gain and increased fasting plasma glucose, homeostasis model assessment of insulin resistance (HOMA-IR), and low density lipoprotein (LDL) cholesterol concentration [122]. As far as metabolic disturbances are concerned, studies by Bahr et al. [120] and Flowers et al. [121] microbiota alteration during psychotropic treatment may correlate with weight gain.

We previously demonstrated that SGA-induced dysbiosis may potentially result in body weight and metabolic disturbances with low-level inflammation and decreased energy expenditure involved in the mechanism. However, since the majority of studies were conducted in rodent models with a high number of unclear risk of bias assessments, these findings need to be considered cautiously and may not be fully replicated in humans [123]. More studies regarding the involvement of psychiatric medications on gut microbiota composition in humans are warranted and, ccurrently, our research team undertook this effort.

The first antidepressant—Iproniazid—Via producing isonicotinoyl radicals may interrupt the bacterial cell cycle and inhibit their growth [124,125]. Tricyclic antidepressants were found to possess anti-plasmid activity and inhibit the growth of *Escherichia coli* Castellani and Chalmers, *Yersinia enterocolitica* Schleifstein & Coleman [126] and *Giardia lamblia* Kofoid & Christiansen [127] by means of decreasing the activity of DNA gyrase [128]. Tricyclic antidepressants are active relative to Plasmodium falciparum [129] and *Leishmania* spp. [130]. Selective serotonin re-uptake inhibitors may inhibit the growth of *Staphylococcus*, *Enterococcus* [131,132,133], *Citrobacter* spp, *Pseudomonas aeruginosa* Migula, *Klebsiella pneumoniae* Trevisan, *Morganella morganii* Brenner et al., *Clostridium perfringens* Hauduroy et al., and *Clostridium difficile* Prevot [131,134]. Efflux pump inhibition may be involved in these properties [135]. Ketamine may control the growth of *Staphylococcus aureus* Rosenbach, *Staphylococcus epidermidis* Evans, *Entercoccus faecalis* Schleifer and Kilpper-Bälz, *Streptococcus pyogenes* Rosenbach, *Pseudomonas aeruginosa* Migula and *Candida albicans* (C.P.Robin) Berkhout [134,136].

## 7. Modulation of Microbiota-Gut-Brain Axis as a Promising Tool to Manage Gastrointestinal and Mental Health

Mood disorders and depression are associated frequently with other GI conditions such as liver disease, inflammatory bowel disease (IBD), food intolerance, enteropathies (e.g., celiac disease), and cancer. Recently, Felice and O’Mahony [137] explained the origin of the high coincidence between stress-related psychiatric conditions and GI symptoms. Dysbiotic gut microbiota via synthesizing neuroactive metabolites are able to counteract secretion, motility, and blood flow within the GI tract and, additionally, transfer neural signals through vagus nerve and spinal cord routes. Therefore, the modulation of microbiota-gut-brain pathways opens up new avenues for the management of chronic diseases both of psychiatric and organic origin [12,138,139,140,141,142,143].

The new treatment avenues could be addressed through the modulation of the microbiota-gut-brain axis by means of prebiotic and probiotic administration. The World Gastroenterology Organization (WGO) recently issued a Global Guideline on Prebiotics and Probiotics use by healthcare professionals [144]. Among probiotics, those are recommended in the management of disorders of gut-brain interaction commonly known as FGIDs: *Lactobacillus plantarum* Bergey et al. 299V, *Bifidobacterium infantis* Reuter 35624, *Bifidobacterium animalis* Scardovi and Trovatelli DN-173 010, and *Saccharomyces boullardii* Henri Boulard CNCM I-745.

### Psychobiotics—New Kid on the Block

Psychobiotics are a new class of probiotics which, when ingested, confer mental health benefits through interactions with the microbiota-gut-brain axis [145]. This term should also include prebiotics, which favorably influence the growth of beneficial gut bacteria [146]. Some of the properties of probiotics are prevalent among different strains, e.g., improvement of the intestinal epithelium renewal while the others are strain-specific, e.g., modulation of the CNS function [147]. Misra et al. noted that psychobiotic strains may be involved in the neuroactive substances synthesis, activate directly neural pathways, modulate neurotrophic factors, protect microbiota against stress, and others [148].

The action of psychobiotics was confirmed in mechanistic studies. For example, Ait-Belgnaoui et al. [149] demonstrated that psychobiotics may significantly reduce stress-induced neuronal activation in three brain regions i.e., hypothalamus, amygdala, and hippocampus, and promote the development of dendrites in the cingulate cortex-center of neurogenesis. Another study confirmed that psychobiotics decreased visceral hypersensitivity intensity and such an action correlated with the concentration of stress hormones (noradrenaline, adrenaline, corticosterone) and was possibly regulated by glucocorticoid receptors [150]. In vivo studies also reported that ingestion of psychobiotics improved GI function through the modulation of GBA in animals experiencing maternal separation [151] and *Citrobacter rodentium* infection [152].

Lately, the importance of psychobiotic use in humans has been acknowledged. Diop et al. was the first to demonstrate that probiotic supplementation reduces symptoms of GI disorders caused by stress in healthy people [153]. Messaoudi et al. showed that psychobiotic strains impact positively psychological stress exponents and reduce urinary free cortisol concentration [154]. Steenbergen et al. [155] demonstrated that, after 4 weeks of probiotic supplementation, a statistically significant improvement of aggression and rumination compared to placebo was found. Allen et al. [156] tested the psychobiotic strain to find that it may improve memory and reduce stress while Kato-Katoka [157] who analyzed a group of students during the exam session reported that the incidence of abdominal pain and cold as vegetative stress symptoms was significantly lowered in the group of students receiving probiotics compared to the placebo group. A recent study by Lv et al. [158] summarized that psychobiotics improve the function of the GI tract in patients with schizophrenia.

In addition, meta-analyses have shown that probiotic intervention may positively affect the mood in healthy persons as well as depressive patients [159]. McKean et al. [160] found that probiotic consumption may diminish the symptoms of depression, anxiety, and stress. Surprisingly, the latest meta-analysis concluded that such an intervention may significantly improve the mood of patients with mild to moderate depression but not the mood of healthy individuals [161]. Consequently, psychobiotics and neurobiotics, which are capable of GBA and HPA modulation, could be advocated in the management of patients endangered with iatrogenic complications associated with pharmacotherapy and polypharmacy. However, more studies—Especially of the highest evidence level—Are necessary to elucidate the psychobiotic potential to improve GBA function.

The use of these new probiotic compounds could be of great use in the daily management of stress and depression in FGIDs patients but also with other GI and extra-intestinal complaints associated with mental or mood alterations [162,163]. The data supporting their use are already strong and new studies that shall create even more evidence to medical societies as well as governmental agencies to help them evaluate the microbiota-gut-brain axis therapeutics in the management of stress in contemporary medicine [164,165]. Table 2 includes examples of psychobiotic strains and their clinical applications [144,148,166]. Since a few probiotic strains were found to be effective to counteract mood disorders and FGIDs, there is still limited data in individuals with ASD, Parkinson’s disease, and Alzheimer’s disease. There is an urgent need for a great investment in clinical trials in these entities [167,168,169,170,171].

## 8. Conclusions

Our review suggests some involvements of GBA deregulation in the origin of disorders of the brain and gut. We hypothesize that stem cell-host microbiome cross talk is potentially involved in GBA disorders. Consequently, molecules mediating GBA signaling may constitute a grip point for the development of diagnostic tools and personalized microbiota-based therapies. In addition, novel treatment protocols based on new compounds interfering with gut derived metabolites as well as small molecules and bioactive lipids playing roles in adult stem cell trafficking are awaiting further developments.

## Figures and Tables

**Table 1:** Microbiota and its metabolites alterations in various psychiatric conditions.

The Disease	Microbiota-Related Fingerprint	Reference
Depression	*↑ Bacteroidetes, Proteobacteria, Actinobacteria, Enterobacteriaceae, Alistipes,* propionic, isobutyric, and isovaleric acids	[60,61,62,63]
	*↓ Faecalibacterium, Bifidobacterium, Lactobacillus*; serotonin, noradrenalin, SCFAs, kynurenic acid, kynurenine	
Schizophrenia	*↑ Corinobacteriaceae, Prevotella, Succinivibrio, Collinsella, Megasphaera, Klebsiella, Methanobrevibacter, Clostridium*	[64,65]
	*↓ Blautia, Coprococcus, Roseburia,*	
Bipolar disorder	*↑ Bacteroides, Actinobacteria, Coriobacteria*	[66,67]
	*↓ Faecalibacterium, Roseburia, Alistipes,*	
Parkinson’s disease	*↑ Bacteroides, Roseburia*	[68]
	*↓ Blautia, Coprococcus, Dorea, Oscillospira, Akkermansia*	
Autism Spectrum Disorder	*↑ Streptococcus, Clostridiales, Comamonadaceae, Akkermansia, Rhosococcus, Oscillospira, Desulvibrio, Burkholderia, Collinsella, Corynebacterium, Dorea, and Lactobacillus;* acetic and propionic acid, p-cresol, Glutamate*,*	[69,70,71,72,73]
	*↓ Firmicutes, Faecalibacterium, Ruminococcus, Proteobacteria, Fuscobacteria, Verrumicrobia, Bifidobacterium, Neisseria, Alistipes, Bilophila, Dialister, Parabacteroides,* and *Veillonella,* butyric acid, tryptophan, kynurenic acid,	
Attention-Deficit Hyperactivity Disorder	*↑ Actinobacteria (Bifidobacterium* genus*)*	[74]
	*↓ Firmicutes (Clostridiales* order*)*	
Alzheimer’s disease	*↑ Blautia, Phascolarctobacterium, Gemella, E.coli, Shigella, Ps. aueruginosa*	[75,76]
	*↓ Ruminococcaceae, Turicibacteraceae, Peptostreptococcaceae, Clostridiaceae, Mogibacteriaceae,* and the genera *SMB53 (*family*, Clostridiaceae) Dialister, Clostridium, Turicibacter,* and cc115 (family *Erysipelotrichaceae)*	
Multiple sclerosis	*↑ * *Akkermansia muciniphila, Acinetobacter calcoaceticus*	[77]
	*↓ * *Parabacteroides distasonis*	
Anorexia nervosa	*↑ * *Methanobrevibacter smithii*	[78]

**Table 2:** Probiotics strains with documented efficacy in gut-brain axis disorders [144,148,166,172].

Condition	Strain
Anxiety and depression	*Lactobacillus fermentum* NS8 and NS9, *Lactobacillus casei* Shirota, *Lactobacillus gasseri* OLL2809, *Lactobacillus rhamnosus* JB-1, *Lactobacillus helveticus* Rosell -52, *Lactobacillus acidophilus* W37, *Lactobacillus brevis* W63, *Lactococcus lactis* W19 and W58, *Bifidobacterium longum* Rosell-175, *Bifidobacterium longum* NCC3001, *Bifidobacterium longum* 1714, *Bifidobacterium bifidum* W23, *Bifidobacterium lactis* W52, *Lactobacillus plantarum* 299v
Stress	*Lactobacillus casei* Shirota, *Lactobacillus helveticus* Rosell -52, *Lactobacillus plantarum* PS128, *Bifidobacterium longum* Rosell-175, *Lactobacillus gasseri* CP230 *
FGIDs	*Lactobacillus plantarum 299v* (DSM 9843), *Escherichia coli* DSM17252, *Bifidobacterium animalis* DN-173, *Saccharomyces boulardii* CNCM I-745 *Bifidobacterium infantis* 35624, *Lactobacillus rhamnosus* NCIMB 30174, *Lactobacillus plantarum* NCIMB 30173, *Lactobacillus acidophilus* NCIMB 30175, *Enterococcus faecium* NCIMB 30176

* Para-psychobiotic-heat inactivated strain.
